# Enhanced Novel Object Recognition and Spatial Memory in Rats Selectively Bred for High Nicotine Preference

**DOI:** 10.3390/brainsci14050427

**Published:** 2024-04-25

**Authors:** Eren Bekci, Ramazan Can Gokmen, Lutfiye Kanit, Oguz Gozen, Burcu Balkan, Ersin O. Koylu, Aysegul Keser

**Affiliations:** 1 Neuroscience Department, Institute of Health Sciences, Ege University, Izmir 35100, Turkey; 2 Department of Physiology, School of Medicine, Ege University, Izmir 35100, Turkey

**Keywords:** nicotine preference, novel object recognition, spatial memory, selective breeding, novelty seeking

## Abstract

This study examined the influence of genetic background on cognitive performance in a selectively bred high nicotine-preferring (NP) rat line. Using the novel object recognition (NOR), novel location recognition (NLR), and Morris water maze (MWM) tests, we evaluated object memory, spatial memory, and spatial navigation in nicotine-naive NP rats compared to controls. Our results demonstrate that in the NOR test, both male and female NP rats spent more time exploring the novel object (higher discrimination index) compared to sex-matched controls. In the NLR, the discrimination index differed significantly from zero chance (no preference) in both NP males and females but not in controls, indicating enhanced spatial memory in the NP line. During MWM acquisition, the NP groups and control males took a shorter path to reach the platform compared to control females. On the probe trial, the distance traveled in the target quadrant was longer for NP males and females compared to their respective controls, suggesting enhanced spatial navigation and learning in the NP rats. The interesting preference for novel objects and locations displayed by NP rats may indicate a potential novelty-seeking phenotype in this line. These results highlight the complex interplay between genetic factors, cognitive function, and nicotine preference.

## 1. Introduction

Tobacco use is a major global health concern, with approximately 1.1 billion people worldwide using tobacco products. Smoking is the most common form of tobacco use and is estimated to cause over 8 million deaths annually, making it one of the leading preventable causes of death globally.

Nicotine, the primary psychoactive component of tobacco, exerts its addictive effects by binding to nicotinic acetylcholine receptors in the brain, leading to the release of neurotransmitters such as dopamine, which is associated with reward. This neurochemical cascade reinforces smoking behavior and results in dependence in many users. Addiction to nicotine is attributed to the presence of drug-context and drug-cue associations. These associations trigger drug-seeking behavior and affect cognition during abstinence, resulting in relapse. A better understanding of the effects of nicotine on learning and memory may facilitate the development of treatments for nicotine addiction [1].

Beyond its addictive properties, nicotine has complex effects on cognitive function. While acute nicotine exposure can improve attention, memory, and cognitive performance in some individuals, chronic use can lead to tolerance and dependence, ultimately impairing cognitive function. Long-term nicotine exposure has been linked to alterations in brain structure and function, particularly in regions involved in learning, memory, and decision making [2,3]. Animal studies have shown that nicotine administration can improve performance in spatial learning and memory tasks such as the Morris water maze (MWM), which assesses the ability to learn and remember the location of a hidden platform in a pool. Using oral nicotine self-administration and the water maze learning test, Nesil et al. showed that chronic nicotine intake preferred by rats facilitated their acquisition of place learning in the task [4]. They also showed that chronic nicotine exposure modified the strategy that female rats used to solve a problem. Another study showed that nicotine administration improved performance on a task requiring human participants to remember a series of numbers in reverse order, indicating an improvement in working memory [5].

Object recognition and location memory can be evaluated through tasks such as novel object recognition (NOR) and novel location recognition (NLR) tests [6,7,8,9]. The hippocampus plays a significant role in object-in-place and recency recognition memory, interacting with brain regions such as the perirhinal or medial prefrontal cortices [10,11]. Studies have also investigated the role of different brain regions such as the anterior cingulate cortex in novel object and location recognition behavior [12]. Object recognition and location memory can be influenced by various factors, including nicotine [13,14]. Acute nicotine administration was shown to enhance novel object recognition and location memory [15]. On the other hand, Kenney et al. reported that acute nicotine treatment enhanced spatial object recognition whereas it impaired novel object recognition [13].

Genetic variants in genes related to nicotine metabolism (*CYP2A6*, *FMO3*, and *UGT2B7*) and nicotinic cholinergic receptors (*CHRNB3*-*CHRNA6*, *CHRNA5*-*CHRNA3*-*CHRNB4*, and *CHRNA4*) have been associated with nicotine dependence [16,17]. Genetic variants also influence the cognitive effects of nicotine. Nicotinic acetylcholine receptor subunits, such as α4, β2, and α7, and variants of genes like *CHRNA5*/*A3*/*B4* have been identified as key players in the cognitive enhancing effects of nicotine [5,18,19]. Genetic factors interacting with nicotine withdrawal can impact cognitive function, as evidenced by research demonstrating disrupted cognitive function in smokers experiencing nicotine withdrawal [20]. Genetic variations affecting the cholinergic and dopaminergic neurotransmitter systems can influence performance on cognitive tasks and the impact of nicotine [21]. Furthermore, nicotine binding sites vary among different inbred mouse strains, suggesting that genetic variability may alter nicotinic acetylcholine receptor binding in different brain regions [22]. Therefore, genetic background may lead to differences in reward and cognitive performance among strains. Genetic variants that modify cholinergic function may result in changes in sensitivity to nicotine’s effects on both the reward system and on learning and memory [23]. 

Golding et al. studied the transgenerational effects of smoking and found that smoking in pre-puberty in grandparents increased fat mass in grandchildren [24]. In another study, maternal grandmother smoking was associated with increased odds of grandchildren having persistent asthma [25]. Maternal nicotine exposure also resulted in behavioral changes, neuropharmacological changes, and epigenetic changes in the F1 and F2 generations of adolescent mice [26]. 

Our laboratory has developed a nicotine-preferring (NP) rat line by selective breeding using a two-bottle choice test consisting of bottles containing tap water with or without nicotine. As a result of this paradigm, rats are categorized into high, median, and low consumption groups (high: 4.02 ± 0.27; median: 3.01 ± 0.25; and low: 2.19 ± 0.18 mg of nicotine/kg). In each generation, the high- and low-consumption groups are selectively bred to form high-preferring and low-preferring rat lines, respectively. High nicotine-preferring breeders of the ninth generation had 7.93 ± 0.69 mg/kg per week [27]. 

Somatic signs and locomotor activity during withdrawal were examined in these high and low NP rats in the eighth generation [28]. During nicotine exposure, the high NP line was more active than the low NP line. During withdrawal, locomotor activity decreased in both lines but decreased more significantly in the high NP line. Somatic signs of withdrawal also increased during nicotine withdrawal [28]. 

In another study, 18^th^-generation high NP rats were tested for alcohol consumption [29]. The animals were given intermittent access to 20% ethanol and tap water using the two-bottle choice procedure with three 24-h ethanol availability periods per week for 6 weeks. The high NP rat line consumed significantly more ethanol compared to controls and preferred ethanol over water. In addition, ethanol consumption was higher in females than males. After a one-week break, the same animals were tested for nicotine preference with oral nicotine self-administration. The results showed that nicotine preference and consumption were greater in the high NP line compared to controls. 

Bayoglu et al. assessed anxiety-like behavior in the 22nd generation of the NP rat line during basal condition and nicotine treatment using the elevated plus maze, open field, and marble burying tests [30]. In the elevated plus maze, high NP animals spent more time in the open arm, preferred being in the open arm, and had longer latency to enter the closed arms compared to controls. In the open field test, the time spent in the central zone was higher for NP rats. In the marble burying test, high NP animals buried fewer marbles than controls. All these results obtained by the elevated plus maze, open field, and marble burying tests are consistent with each other and suggest decreased anxiety-like behavior in the high NP line both during basal condition and oral nicotine treatment [30].

The present study was performed to assess novel object memory and spatial memory in the high NP rat line. Male and female high NP rats naive to nicotine were subjected to the NOR, NLR, and MWM tests. The genetic background underlying increased nicotine preference in this rat line may also regulate cognitive performance. Our hypothesis was that the genetic background of this rat line would cause a deficit in cognitive performance, such that high NP rats might increase their nicotine consumption in order to restore or improve their cognitive performance. This is the first study to examine the cognitive performance of a rat line selectively bred for oral nicotine preference.

## 2. Materials and Methods

### 2.1. Animals and Housing

A total of 20 nicotine-naïve adult (3–4 months old), selectively bred NP rats (10 male and 10 female) and 24 adult male and female Sprague Dawley rats (12 male and 12 female) were obtained from the Ege University Animal Breeding Facility.

The NP rat line was developed in our laboratory by selective breeding using the two-bottle choice method, as described previously [27,28,29]. Briefly, animals were housed in same-sex groups with ad libitum access to food and water while they were not being used for selection or breeding. During selection, each animal was caged individually and two bottles containing tap water with or without nicotine (20 mg/L free base) were installed on every cage, allowing the animals to have a free choice to consume nicotine during testing for 6 weeks. Each week, total water and nicotine consumption was measured and Ward analysis was used to select the high NP rats which would be used as breeders for the next generation. This procedure is repeated for each generation [27,28,29]. The NP animals used in this study are the 27th generation (F27) of this rat line and had never been exposed to nicotine.

During this study, rats were kept in standard plastic cages (3–4 rats/cage) with food and water provided ad libitum, under standard laboratory conditions (12 h light–12 h dark cycle, lights on 07:00–19:00, and 20–22 °C). All procedures were approved by Ege University Animal Ethics Committee (2012-074) and carried out in compliance with the European Communities Council Directive (2010/63/EU) regarding the use of animals for scientific purposes. Every effort was made to minimize animal suffering and reduce the number of animals used.

### 2.2. Behavioral Experiments

**Figure 1:**
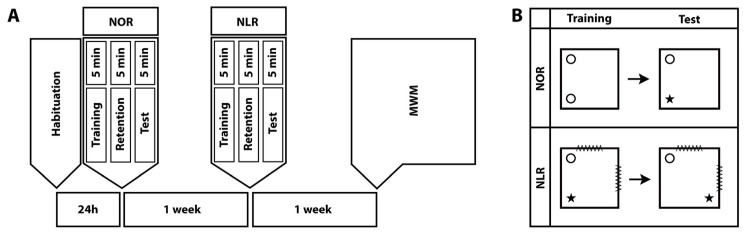
Timeline of the behavioral experiments. NOR: Novel object recognition, NLR: Novel location recognition, and MWM: Morris water maze (**A**). The experimental design for the NOR and NLR tests (**B**).

The animals were subjected to NOR, NLR [6,24], and MWM tests, in the order stated, with one-week intervals between the tests (Figure 1A).

### 2.3. Novel Object Recognition Test

The NOR test is designed to assess short- or long-term recognition memory in rodents and relies on the innate preference of rodents for novelty [31,32]. It consists of habituation, training, and test sessions (Figure 1B). The same chambers were used for NOR and NLR testing. Their dimensions were 70 cm wide × 70 cm long × 45 cm high and they had a black bottom and cream-colored walls. Video cameras were suspended 110 cm above the test arena. During habituation, each animal was allowed to freely roam the chamber for 5 min. In the training session, held 24 h after habituation, the animals were exposed for 5 min to two identical objects (A1 and A2) placed in two adjacent corners of the test arena. The amount of time the animal devoted to “exploring” the objects (sniffing the object or tilting the head at a distance less than 2 cm from the object) was measured. During the retention interval, which lasted 5 min, the experimenter removed both objects and replaced one with an identical copy (A) and the other with a new object (B). In the test session, the amount of time the animals devoted to exploring the objects (A and B) was recorded for 5 min [31,32,33]. To avoid object and position bias, both the objects and their positions in the chamber were randomized. The objects were different enough for animals to distinguish between but not so different as to make them avoid one object. Both the objects and the arena were cleaned with 70% ethanol after each animal to avoid any odor cues for the next animal. The total exploration time was calculated by adding the times spent exploring the novel and familiar objects. The discrimination index was calculated by dividing the difference in time devoted to each object by the total exploration time ([time spent exploring the novel object − time spent exploring the familiar object]/total exploration time). The calculated discrimination index values vary between −1 and +1 and zero is the fixed chance level. A positive discrimination index value indicates that the animal spent more time investigating the novel object. On the other hand, a discrimination index of 0 indicates an equal amount of time spent around familiar and novel objects.

### 2.4. Novel Location Recognition Test

NLR is an important form of spatial memory, comprising different subcomponents that each process specific types of information within memory, such as remembering objects, remembering the positions of objects, and linking this information in the memory [34]. The NLR test consisted only of training and test sessions; there was no habituation session as it was conducted in the same chambers used for NOR testing (Figure 1B). For the duration of the NLR tests, visual cues were placed on the chamber walls to help the animals locate and determine their routes. In the training session, each animal was exposed to two different objects and in the test session, the position of one of the objects was changed [35]. The training and test sessions were each separated by a 5-min retention interval (Figure 1). The amount of time the animals devoted to exploring each object was measured and the total exploration time and discrimination index of the relocated vs. unmoved object were calculated as described above for the NOR test.

### 2.5. Morris Water Maze

The MWM test was carried out in a circular pool with a diameter of 130 cm and height of 75 cm, located in a 3 m × 4 m room. The pool was filled with water to a depth of 45 cm. The water was at a temperature of 21 °C and made opaque with a non-toxic dark yellow dye [4]. A 12 cm × 12 cm platform placed in the southwest (SW) quadrant was used as a target for the animals to get out of the water. Acquisition training was carried out for 6 days. The water maze tank was virtually divided into south (S), west (W), north (N), and east (E) quadrants. On the first day, the platform was visible and 2 cm above the water level. Before being released into the water for the first time, each animal was placed and held on the platform for 30 s. They were then released into the water once from each quadrant in the order of SWNE while the platform was visible. Starting from the second day, the platform was hidden 2 cm below the water level. On the second day, each animal was again placed on the platform for 30 s before swimming sessions. Between day 2 and day 6, the animals were released into the water once from each quadrant in the order of WNES, NESW, ESWN, SWNE, and WNES, respectively. After finding the platform, the animals were allowed to remain on it for 15 s. If the animal was not able to find the platform within 60 s, the examiner (E.B.) guided the animal to swim to the platform and allowed it to stay on the platform for 15 s. On day 7 (probe trial), the platform was removed from the pool. The animals were released from the quadrant opposite where the platform was located before it was removed. 

The experiments were recorded on video and an open-source animal tracking software [36] was used to measure the path length (cm) animals took to find the platform for each release [4,37,38]. Between days 1 and 6, the total path length to reach the platform was measured and the daily performance was calculated by averaging the values obtained from each quadrant. On day 7, the distance animals traveled in the SW quadrant (target quadrant) was recorded.

### 2.6. Statistical Analysis

The data were evaluated using the IBM SPSS v25.00 and GraphPad Prism v5.01 statistical software. Normality was checked by using the D’Agostino-Pearson normality test. Univariate analysis of variance (ANOVA) was performed with line (control/NP) and sex (female/male) as between-subjects factors. If a main effect of a between-subjects factor was detected in univariate ANOVA, one-way ANOVA with a post hoc Duncan test was performed. Repeated measures ANOVA was performed with object (novel/familiar for NOR, relocated/unmoved for NLR) as a within-subjects factor and line (control/NP) and sex (female/male) as between-subjects factors. One-sample t-tests were also used to compare discrimination indexes against the zero chance level, where zero represents no preference. In addition, any outliers in the data sets were identified using the Grubbs test (designed to detect a single outlier in data showing normal distribution) and removed from the analysis [39]. Animals with a total investigation time of less than 20 s were excluded from the analysis [40]. 

The paths taken by animals to reach the platform in the MWM experiments were analyzed using repeated-measures ANOVA with days as the within-subjects factor and line and sex as between-subjects factors. Repeated-measures ANOVA was also performed using groups as a between-subjects factor, followed by post hoc Duncan tests. Performance on each day was also analyzed using univariate ANOVA with line and sex as between-subjects factors. If a main effect of a between-subjects factor was detected in univariate ANOVA, one-way ANOVA analysis with a post hoc Duncan test was performed.

## 3. Results

### 3.1. Novel Object Recognition

**Figure 2:**
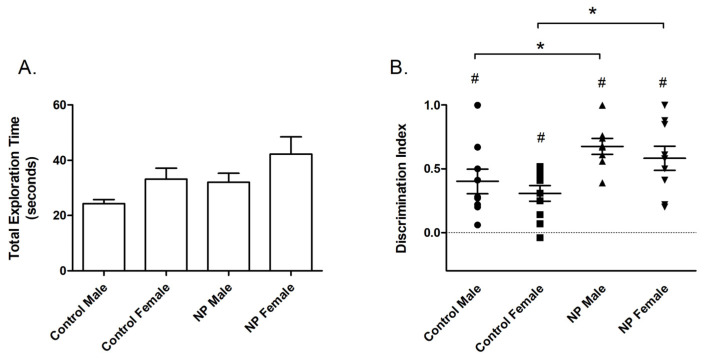
Novel object recognition. Total exploration time (mean + SEM) during the training session (**A**). Discrimination index (mean + SEM) for the test session (**B**). * Significant difference from controls (*p* < 0.05). **#** Significant difference from zero chance level (*p* < 0.005). n = 8–11 for each group.

The results of NOR testing are shown in Figure 2. According to univariate ANOVA analysis of total exploration time during the training session, sex had a main effect (F_(1,38)_ = 5.128; *p* = 0.03) (Figure 2A). The total exploration time was longer in females than males. One-way ANOVA revealed a significant difference between groups (F_(3,38)_ = 2.983; *p* = 0.044). The post hoc Duncan test showed a significant difference between control males and NP females (*p* < 0.05, not shown in the figure).

In the test session, line had a main effect on the discrimination index (F_(1,35)_ = 11.615; *p* = 0.002) in univariate ANOVA (Figure 2B). Animals from the NP line spent more time with the novel object and had a higher discrimination index than controls. A significant difference between the study groups emerged in one-way ANOVA (F_(3,35)_ = 4.324; *p* = 0.011). According to post hoc analysis, discrimination index values were higher for high NP males and high NP females when compared with sex-matched controls (*p* < 0.05 for both, Figure 2). One-sample t-tests showed that discrimination indexes were significantly different from the zero chance level in all groups (control males: *p* = 0.003; control females: *p* = 0.001; high NP males: *p* < 0.001; and high NP females: *p* < 0.001).

**Figure 3:**
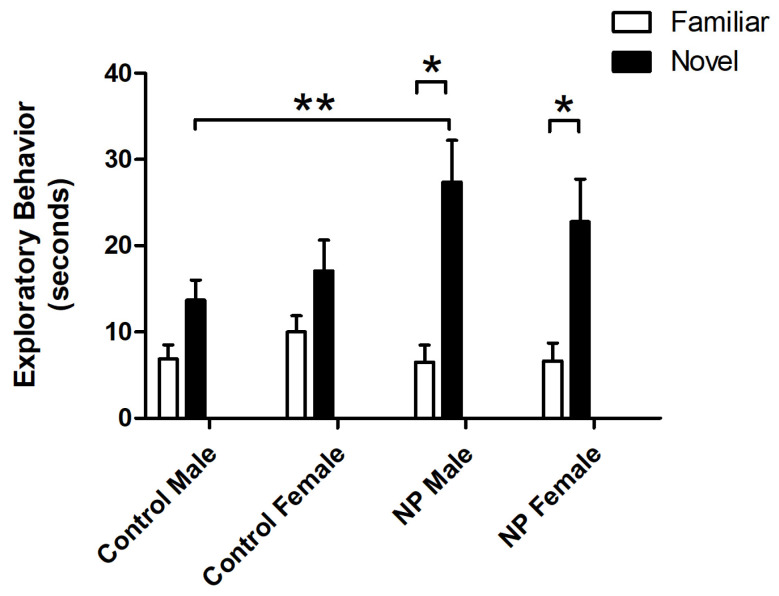
Exploration times for familiar and novel objects in the novel object recognition test session. * Significant difference between novel and familiar objects in the NP groups (*p* < 0.05). ** Significant difference in novel object exploration times between NP and control males (*p* < 0.05). n = 8–11 for each group.

Object exploration behavior was also analyzed by repeated-measures ANOVA. There was a main effect of object (F_(1,33)_ = 66.750; *p* < 0.0001), with animals exploring the novel object for a longer time. A significant interaction between object and line was observed (F_(1,33)_ = 13.793; *p* = 0.001). One-way ANOVA results revealed a significant difference between the study groups (F_(7,77)_ = 3.771; *p* = 0.002). In post hoc analysis, NP males explored the novel object significantly more than control males (*p* < 0.05), while control and NP females did not show any significant difference. Both NP males and females explored the novel object significantly more than the familiar object (*p* < 0.05 for both, Figure 3).

### 3.2. Novel Location Recognition Test

**Figure 4:**
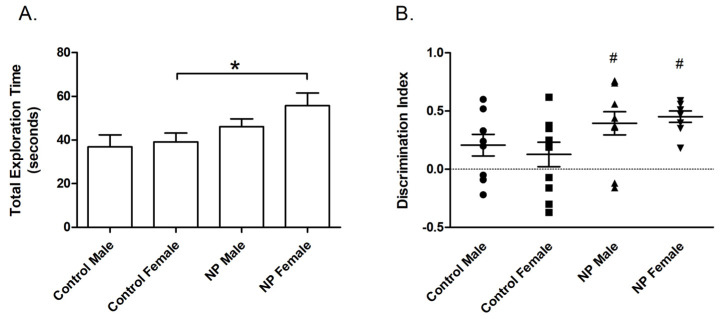
Novel location recognition. Total exploration time (mean + SEM) during the training session (**A**). Discrimination index (mean + SEM) for the test session (**B**). * Significant difference from controls (*p* < 0.05). # Significant difference against the chance level of zero (*p* < 0.005). n = 8–11 for each group.

In univariate ANOVA, there was a main effect of line (F_(1,38)_ = 7.450; *p* = 0.010) on the total exploration time in the training session of the NLR test. The NP animals spent more time exploring the objects. One-way ANOVA revealed a significant difference between groups (F_(3,37)_ = 3.102; *p* = 0.039). The total exploration time was significantly higher in the NP female group than in control females (Figure 4A).

Line also had a main effect (F_(1,36)_ = 7.597; *p* = 0.009) on the discrimination index in the testing session according to univariate ANOVA. One-way ANOVA and post hoc tests showed no difference in the discrimination index between controls and high NP animals. However, preference for the relocated object was significantly different from zero chance (no preference) in NP males and females (*p* = 0.003 and *p* < 0.0001, respectively), whereas a similar effect was not observed in control groups (Figure 4B). Since the two control groups were not different from each other (*p* > 0.05), the one sample t-test was repeated by omitting sex as a factor. The results showed that the discrimination index of controls was significantly different from the chance level of zero (*p* = 0.029).

**Figure 5:**
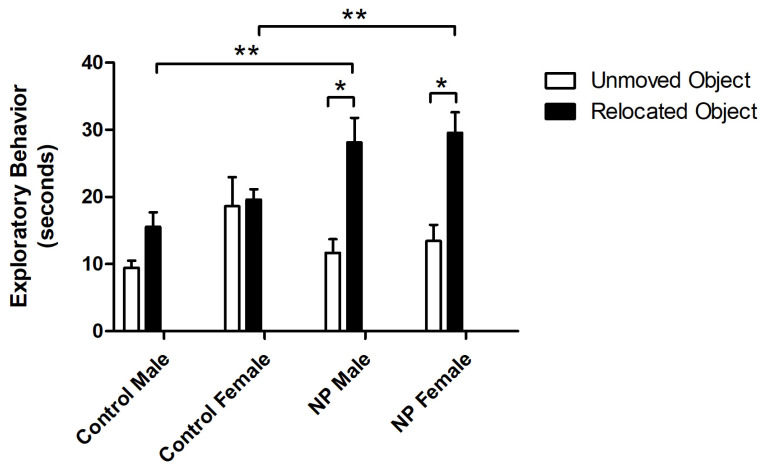
Exploration times of relocated and unmoved objects in the novel location recognition test session. * Significant difference between relocated vs. unmoved objects in the NP groups (*p* < 0.05). ** Significant difference in novel location exploration times from controls (*p* < 0.05). n = 8–11 for each group.

In the test session, exploratory behavior toward relocated and unmoved objects was analyzed by repeated-measures ANOVA. The main effects of object and line were significant (F_(1,34)_ = 26.580; *p* < 0.0001, F_(1,34)_ = 5.916; and *p* = 0.02, respectively) (Figure 5). There was also a significant interaction between object and line (F_(1,34)_ = 10.923; *p* = 0.002). One-way ANOVA showed a difference between groups (F_(7,75)_ = 6.887; *p* < 0.0001). In both NP male and female groups, the preference for the relocated object was higher compared to sex-matched controls (*p* < 0.05 for both). Both NP male and female animals preferred the relocated object compared to the object that remained in the same place (*p* < 0.05 for both).

### 3.3. Morris Water Maze 

**Figure 6:**
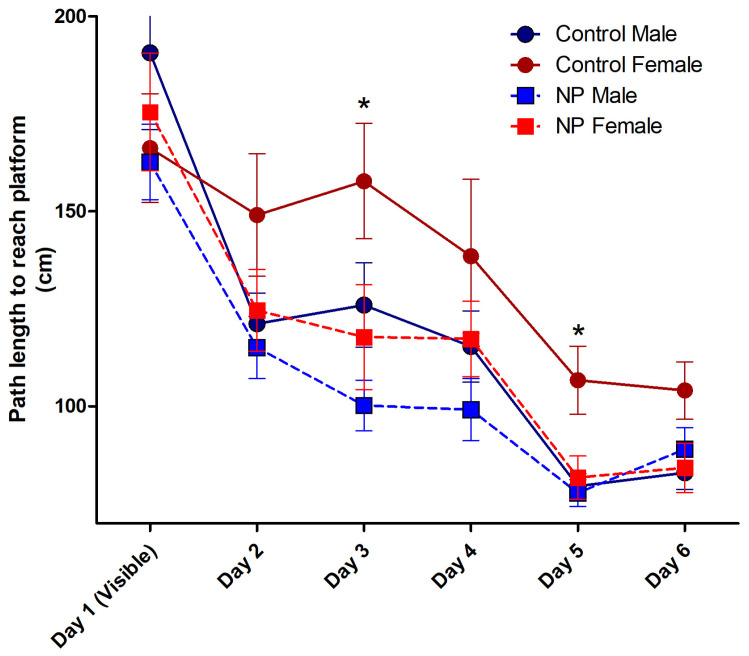
The path length to reach the platform during acquisition training in the Morris water maze. Over the 6 days, control females took a longer path to the platform compared to the other groups (*p* < 0.05, not marked in the figure). On day 3, both NP males and females took a shorter path than control females (* *p* < 0.05). On day 5, all other groups took shorter paths than control females (* *p* < 0.05). n = 10–12 per group for each day.

Repeated-measures ANOVA of the path taken to reach the platform for the first six days of the MWM test revealed the main effect of day (F_(5,36)_ = 29.161; *p* < 0.0001). Line (F_(1,40)_ = 7.534; *p* = 0.009) and sex (F_(1,40)_ = 5.425; *p* = 0.025) were significant between-subjects factors. The path to find the platform decreased by day in NP animals and males. There was no interaction between the factors (Figure 6).

After elucidating the effects of line and sex, repeated-measures ANOVA analysis was performed using group as the between-subjects factor. The main effect of groups was significant (F_(3,40)_ = 4.600; *p* = 0.007). Control females took a longer path to the platform compared to other groups (*p* < 0.05). The high NP female and male groups exhibited a learning pattern similar to that of the control males (Figure 6).

When each day was analyzed separately using univariate ANOVA, a significant difference was found on days 3 and 5. On day 3, sex (F_(1,40)_ = 4.118; *p* = 0.049) and line (F_(1,40)_ = 7.328; *p* = 0.010) had a significant effect without any interaction between them. One-way ANOVA (F_(3,40)_ = 4.015; *p* = 0.014) and post hoc analysis showed that NP males and NP females took a shorter path than control females. There was no difference between control males and control females or between NP males and NP females. On day 5, sex (F_(1,40)_ = 6.929; *p* = 0.012) and line (F_(1,40)_ = 5.135; *p* = 0.029) had a significant effect without interaction. One-way ANOVA (F_(3,40)_ = 5.651; *p* = 0.003) followed by post hoc analysis showed that the paths taken by control males, NP males, and NP females were shorter than control females (*p* < 0.05 for all).

**Figure 7:**
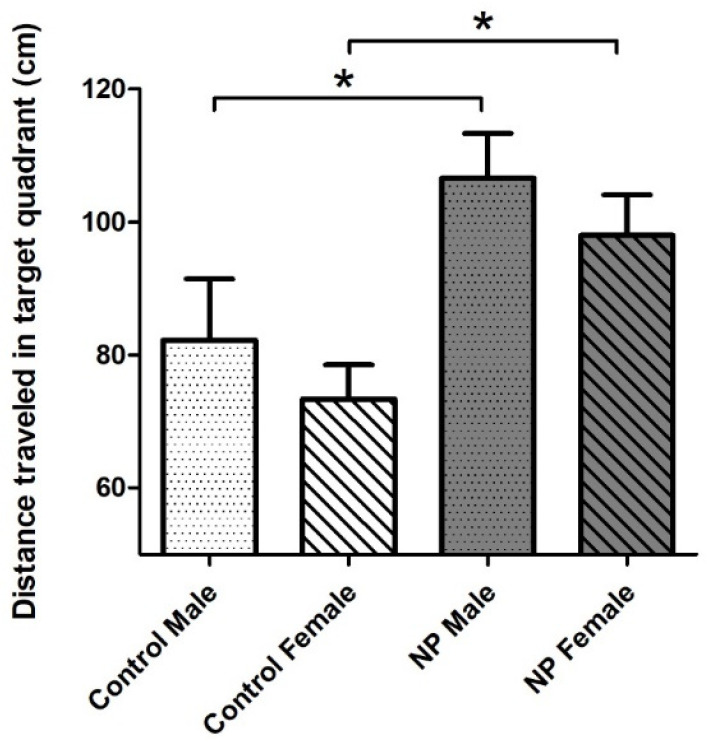
Distance traveled in the target (SW) quadrant on probe trial (day 7) of the Morris water maze test. NP males and NP females swam a longer distance in the target quadrant compared to sex-matched controls (* *p* < 0.05). Data are given as mean + SEM. n = 10–12 for each group.

When the probe trial (day 7) results were examined with univariate ANOVA, the main effect of line emerged (F_(1,40)_ = 11.879; *p* = 0.001). Sex did not have a significant effect. There was a significant difference among the groups according to one-way ANOVA (F_(1,40)_ = 4.471; *p* = 0.008). NP males and NP females swam a longer distance in the target quadrant compared to sex-matched controls, indicating that they remembered where the platform was previously located and searched for it (Figure 7).

## 4. Discussion

This study demonstrated increased novel object and novel location preferences as well as enhanced spatial learning and memory in nicotine-naive rats from a high NP rat line. Therefore, the behaviors displayed by the high NP rats on cognitive tests are a reflection of their genetic background, not an effect of nicotine treatment. The high NP animals used in this study belong to the 27th generation of this selectively bred rat line. Previous generations were selected based on their oral nicotine preference and the offspring of the 26th generation were used in this study. In other words, the rats used in the study are a result of selection for high oral nicotine preference and consumption across 26 generations. Our previous studies showed that offspring of this rat line increased their nicotine consumption from generation to generation [28]. The 8th and 18th generations preferred oral nicotine more than a low-NP rat line [28] and control Sprague Dawley rats [29]. We hypothesize that the high nicotine preference demonstrated by this rat line stems from genetic variations that are associated with the neurotransmitter systems regulating the mesocorticolimbic (reward) system.

The NOR and NLR tests are used to assess memory for object identity and location, respectively [6,41,42]. In this study, the novel object discrimination indexes for high NP males and females were higher than those of control males and females, respectively. This means that they spent significantly more time with the novel object compared to the familiar object. While control animals also spent more time with the novel object than the familiar object, the difference did not reach statistical significance. Also, high NP males explored the novel object more than control males. In the NLR test, there was no significant difference in the discrimination index of novel location between the high NP and control groups. However, the novel location discrimination indexes for both male and female high NP rats were significantly different from chance, whereas no difference was observed in control animals. This indicates that high NP males and females spent significantly more time exploring the relocated object than the unmoved object. Again, control animals also explored the relocated object more but not significantly longer than the unmoved object. Additionally, in the acquisition training of the MWM test, high-NP females took a shorter path to find the platform compared to control females, while there was no difference between high-NP males and control males. In the probe trial, both NP males and females searched the target quadrant more than control males and females, respectively. The MWM results show that female NP animals, in particular, acquired spatial memory faster and both male and female NP animals were better at spatial navigation compared to sex-matched controls. In contrast to our hypothesis that the genetic background of this rat line would cause a deficit in cognitive performance, all of these findings suggest enhanced object memory, location memory, and spatial navigation in high NP males and females.

The cholinergic, dopaminergic, and glutamatergic neurotransmitter systems play interrelated roles in reward and cognitive processes such as memory and learning [43,44,45,46,47]. Genetic variations in the receptors and/or enzymes involved in the synthesis or metabolization of these neurotransmitters can impact both reward-related behaviors and spatial learning. The genetic background that influences nicotine reward in the high NP rat line may interact to shape their cognitive functions (object recognition and spatial learning). The genetic features of this selectively bred rat line have not yet been assessed. However, the cholinergic system is a possible candidate. The α4β2 nicotinic receptors mediate nicotine-induced reward and play a key role in the development of nicotine addiction. There is high expression of α4β2 receptors in the mesocorticolimbic reward system [47]. Nicotine self-administration is impaired in α4- and β2-knockout mice [48,49]. On the other hand, rodent studies indicate that the medial prefrontal cortex (anterior cingulate, prelimbic, and infralimbic cortices), hippocampus, perirhinal, entorhinal, and retrosplenial cortices, and anterior thalamus play important roles in the recognition of objects and their location [50]. These brain regions also express high levels of α4β2 receptors [51,52]. The activation of nicotinic receptors in the perirhinal and hippocampal cortices facilitate object recognition and location memory [15] and the activation of α4β2 receptors in the medial prefrontal cortex enhances object recognition memory [53]. Nicotine was also shown to enhance object recognition memory through the inhibition of voltage-dependent potassium 7 channels in the medial prefrontal cortex of mice [53]. In addition, α4β2 antagonist injection into the hippocampus impairs spatial memory in the eight-arm radial maze [54] and β2-knockout mice show impaired spatial discrimination in this maze [55]. In contrast, systemic injection of NS9283, a positive allosteric modulator of α4β2, improved performance on the MWM test [56] and continuous infusion of ABT-089, an α4β2 and α6β2 partial agonist, enhanced spatial learning of aged rats in the MWM [57]. Varenicline, a partial α4β2 and full α7 agonist, also enhanced object recognition in mice [58].

In the statistical analysis of the data recorded during the acquisition training of the MWM test, sex emerged as a significant main effect. Control females traveled a longer path to find the platform compared to the control males, while there was no sex difference between the NP groups. This finding is consistent with previous studies [59,60]. The better navigational skills in males have been attributed to different strategy choices. Female rodents preferred to rely on visual cues, whereas male rodents preferred to rely on navigational (spatial) cues [61,62]. Some authors also suggested that stress associated with the MWM test could interfere with the performance of females [63], while others suggested that estrogen and progesterone impaired acquisition in the MWM [64]. Likewise, in our study, the sex difference observed between control males and females during the acquisition training disappeared on the probe trial. On the other hand, NP females performed better than control females and very similar to NP males both during acquisition training and on the probe trial. The genetic makeup of NP females may modify the behavioral strategy they use during spatial navigation, leading to similar performance in NP females and males.

Novelty seeking is a personality trait characterized by a tendency to seek out novel experiences that evoke strong emotions. This behavioral construct involves novelty preference, reward dependence, and risk taking [65]. The novelty-seeking phenotype predicts drug use in humans [66], with evidence indicating that high novelty-seekers have an increased risk of drug use compared to low novelty-seekers [67]. The novelty-seeking trait is known to be mediated by the mesolimbic dopaminergic system in a similar way to drug-seeking behavior [31]. In rodents, novelty-seeking behavior encompasses the tendency of rodents to explore new environments, stimuli, or experiences [68]. Previous studies reported that selectively bred high responder rats, which display novelty-seeking behavior when exposed to novel environments, recognized the novel object in the NOR test, whereas selectively bred low responder rats did not [69]. It is suggested that novel object recognition ability may reflect individuals’ tendency for novelty seeking. Furthermore, there are studies that suggest that NOR and NLR tests provide a measure of novelty-seeking behavior as well as assessing cognitive performance [12,70]. The two tests rely on the animals’ preference for novelty. In the NOR test, novelty-seeking behavior is inferred based on the amount of time spent exploring the novel object relative to the familiar object. On the other hand, in the NLR test, novelty-seeking behavior is measured based on the amount of time the rodent spends exploring the object in the novel location compared to the object in the familiar location, thus focusing on the spatial aspect of novelty. The NOR and NLR tests performed in this study showed that both male and female high NP animals preferred the novel object and novel location, which supports a novelty-seeking phenotype in this selectively bred rat line. Elevated plus maze and open field tests are used for measuring novelty-seeking behavior as well as anxiety-like behavior in animals [65]. Previous studies showed that high novelty-seeking rodents, compared to the controls, displayed decreased anxiety-like behavior on the open field and elevated plus maze tests [71,72]. Studies using rodents bred for high alcohol preference also report a lower anxiety-like phenotype in the elevated plus maze and open field [73,74]. In our previous study by Bayoglu et al. (2023), similar to the high-novelty seeking rodents reported in the literature, high NP rats displayed reduced anxiety-like behavior in the open field and elevated plus maze tests both before and during chronic nicotine treatment [30]. High NP rats experience reduced levels of anxiety when they are exposed to novel environments such as the test apparatuses used in the NOR, open field or elevated plus maze tests, and display high novelty-seeking behavior. Reduced anxiety levels may affect novel object/location recognition and may reflect high novelty-seeking behavior in this rat line.

Transgenerational effects of nicotine were reported in previous studies [24,25,26]. Locomotor activity and oral nicotine consumption in both the F1 and F2 generations were increased by maternal nicotine exposure. In addition, dopamine transporter function was impaired and dopamine uptake in the striatum and cortex of mice was decreased by maternal nicotine exposure for both F1 and F2 generations. These changes were accompanied by decreased global methylation in the striatum and cortex. Maternal nicotine exposure decreased corticostriatal DNA methyltransferase 3A expression in adolescent F1 and F2 mice and decreased methyl-CpG-binding protein 2 level in the adolescent F1 and F2 mouse frontal cortex and hippocampus [25,26]. These studies suggest that nicotine can induce transgenerational effects through genetic and epigenetic mechanisms.

## 5. Conclusions

This study demonstrated enhanced novel object recognition and spatial memory in a rat line selectively bred for high oral nicotine preference. This phenotypic feature may be a result of their genetic background affecting a common substrate that plays an important role in both the reward system and the neural pathways that regulate cognitive performance. Nicotine exposure in ancestors may influence the offspring through transgenerational effects. Thus, this genetic background may lead to high nicotine consumption and enhanced object memory, location memory, and spatial navigation. Alternatively, the enhanced novel object and novel location preference displayed by NP animals may reflect a novelty-seeking phenotype in this rat line, which leads to increased vulnerability to drug addiction. In this study, we assessed the cognitive performance of nicotine-naive NP rats. In our future studies, we plan to examine the effects of nicotine on cognitive performance in the NP rat line.

## Data Availability

The data presented in this study are available on reasonable request from the corresponding author. The data are not publicly available because dissemination has yet to be explicitly approved by the local ethics committee.
